# Genome-Wide Study of Response to Platinum, Taxane, and Combination Therapy in Ovarian Cancer: *In vitro* Phenotypes, Inherited Variation, and Disease Recurrence

**DOI:** 10.3389/fgene.2016.00037

**Published:** 2016-03-22

**Authors:** Brooke L. Fridley, Taraswi M. Ghosh, Alice Wang, Rama Raghavan, Junqiang Dai, Ellen L. Goode, Jatinder K. Lamba

**Affiliations:** ^1^Department of Biostatistics, University of Kansas Medical CenterKansas City, KS, USA; ^2^Department of Experimental and Clinical Pharmacology, University of MinnesotaMinneapolis, MN, USA; ^3^Department of Health Sciences Research, Mayo ClinicRochester, MN, USA; ^4^Department of Pharmacotherapy and Translational Research, University of FloridaGainesville, FL, USA

**Keywords:** pharmacogenomics, genome-wide association, carboplatin, paclitaxel, ovarian cancer, cell viability, apoptosis, lymphoblastoid cell lines

## Abstract

**Background:** The standard treatment for epithelial ovarian cancer (EOC) patients with advanced disease is carboplatin-paclitaxel combination therapy following initial debulking surgery, yet there is wide inter-patient variation in clinical response. We sought to identify pharmacogenomic markers related to carboplatin-paclitaxel therapy.

**Methods:** The lymphoblastoid cell lines, derived from 74 invasive EOC patients seen at the Mayo Clinic, were treated with increasing concentrations of carboplatin and/or paclitaxel and assessed for *in vitro* drug response using MTT viability and caspase3/7 apoptosis assays. Drug response phenotypes IC50 (effective dose at which 50% of cells are viable) and EC50 (dose resulting in 50% induction of caspase 3/7 activity) were estimated for each patient to paclitaxel and carboplatin (alone and in combination). For each of the six drug response phenotypes, a genome-wide association study was conducted.

**Results:** Statistical analysis found paclitaxel *in vitro* drug response phenotypes to be moderately associated with time to EOC recurrence (*p* = 0.008 IC50; *p* = 0.058 EC50). Although no pharmacogenomic associations were significant at *p* < 5 × 10^−8^, seven genomic loci were associated with drug response at *p* < 10^−6^, including at 4q21.21 for carboplatin, 4p16.1 and 5q23.2 for paclitaxel, and 3q24, 10q, 1q44, and 13q21 for combination therapy. Nearby genes of interest include *FRAS1, MGC32805, SNCAIP, SLC9A9, TIAL1, ZNF731P*, and *PCDH20*.

**Conclusions:** These results suggest the existence of genetic loci associated with response to platinum-taxane therapies. Further research is needed to understand the mechanism by which these loci may impact EOC clinical response to this commonly used regimen.

## Introduction

Epithelial ovarian cancer (EOC) is the fifth leading cause of cancer death among women in the United States (6% of cancer deaths); in 2015, it is estimated that 14,180 women will die from the disease (Siegel et al., 2015). The standard treatment for patients with advanced disease is initial debulking surgery followed by carboplatin-paclitaxel combination chemotherapy (Marsh, 2009). Five-year overall survival remains around 45% (Marsh, 2009), yet there is a wide inter-patient variation in response. Currently there are few reliable prognostic biomarkers for the classification of patients and treatment response.

Platinating agents, such as carboplatin, interfere with DNA via inter-strand, intra-strand, and DNA-protein crosslinks, thereby causing DNA damage and prevention of cell division and growth, resulting in cell-cycle arrest and apoptosis (Dekou et al., 2001). Although platinum-based drugs are widely used in cancer treatment, many tumors are completely resistant to these drugs, and no clinical response is attained. Major molecular mechanisms underlying this resistance might involve alteration in platinum inactivation or reduced intracellular accumulation by uptake/efflux transporters, increased repair of adducts, increased adduct tolerance or failure of apoptotic pathway. Taxane agents, such as paclitaxel, are commonly used chemotherapeutic drugs often in combination with platinating agents. Taxanes block cell division by binding to α-tubulin, stabilizing the microtubules, thus resulting in cell death (Huizing et al., 1995; Jordan and Wilson, 2004). The development of taxane resistance is common, where response has been linked to metabolism and disposition molecules, such as, cytochrome P450s and drug transporters (e.g., *ABCB1, ABCG2, ABCC1, ABCC2*, and *SLC01B3*; Hewett et al., 2002; Rodriguez-Antona, 2010). In particular, studies in colorectal cell lines and tumor tissue have shown that CYP2C8 may play a role in paclitaxel resistance and the CYP3A may be involved in local inactivation of paclitaxel (Martinez et al., 2002; Garcia-Martin et al., 2006). Additionally, *CYP2C8* and *CYP3A4* are high involved in the metabolism of paclitaxel in patients with ovarian cancer (Bergmann et al., 2011), while breast cancer patients carrying the ^*^3 variant of *CYP2C8* have better response to paclitaxel, but at an increase in peripheral neurotoxicity (Hertz et al., 2012).

However, these molecules do not explain all the variation in taxane response or resistance. Inherited variation in many of the genes encoding these molecules have been assessed for association with clinical outcome with inconsistent results (Peethambaram et al., 2011; Johnatty et al., 2013; White et al., 2013); genome-wide searches to date have also failed to identify variants associated with outcome after correction for genome-wide testing (*p* < 5 × 10^−8^). Patient-derived cell line based model systems represent a novel way to identify genomic predictors of drug response. Although lymphoblastoid cell lines (LCLs) derived from participants in the international HapMap project have been used to identify genomic predictors of cytotoxic effects of various chemotherapeutic agents (Li et al., 2008, 2010; Niu et al., 2010; Huang et al., 2011; Wu et al., 2011), they are limited as they are not derived from the EOC population but from healthy individuals.

In this study, we generated LCLs derived from Mayo Clinic EOC patients, conducted *in vitro* cytotoxic studies, and associated *in vitro* drug response phenotypes with germline genotype. Utilizing patient-derived LCLs, as opposed to commercially available LCLs, allows us to screen and directly correlate *in vitro* phenotypes and clinical responses. These genome-wide association scans (GWAS) should contribute to the identification of predictive markers of treatment responses and ultimately improve clinicians' ability to tailor therapy decisions for EOC patients.

## Materials and methods

### Patients, lymphoblastoid cell lines, and cytotoxicity assays

Prior to initiation of chemotherapy, ovarian cancer patients diagnosed at the Mayo Clinic between 2000 and 2003 provided blood for immediate germline DNA extraction and for the creation of Epstein Barr Virus (EBV)-transformed LCLs. Samples from 74 patients were successfully transformed and subjected to *in vitro* drug testing. All patients provided informed written consent, including for passive and active follow-up, using protocols approved by the appropriate Institutional Review Board at the Mayo Clinic in Rochester, MN.

*In vitro* cellular chemo-sensitivity studies of LCLs were performed in two batches (*N* = 33, *N* = 41) using identical procedures and assays. Cells were maintained in RPMI1640 media supplemented with 2 mM L-glutamine, and 15% fetal bovine serum at 37°C under 5% CO_2_. Following 24 h incubation, LCLs were treated with increasing concentrations of carboplatin and/or paclitaxel (in duplicate). The concentrations of carboplatin were 0, 5, 10, 20, 40, 80, and 128 μM, while for paclitaxel were 0, 4.5, 7.5, 10, 20, 40, and 80 nM when used as single agents. In drug combination experiments, we used half of the doses for each drug in increasing doses (i.e., 2.5 μM carboplatin + 2.25 nM paclitaxel for “dose level 1” and so on). Cell viability 48 h post-treatment was determined using standard MTT assay (Li et al., 2008; Gamazon et al., 2010). Caspase3/7 (Promega) apoptosis assays were performed at the same time in parallel plates. A Synergy 3 plate reader (BioTek Instruments) was used to read absorbance (cell viability using MTT) or fluorescence (for caspase3/7 activity) intensities.

Four parameter logistic dose response curves $Y_{i}\;=\;\alpha +(\beta -\alpha )∕\left(1+{\left[\frac{D_{i}}{\theta }\right]}^{\phi }\right)$ were fit to the *in vitro* drug response measurements (cell survival and caspase3/7 activity assays) for each LCL and treatment (paclitaxel, carboplatin, and combination), where *Y*_*i*_ is the measurement at dose *i* (*Di*), α α is the estimated bottom of the curve (i.e., measurement as *Di* → ∞), β → ∞), β is the estimated top of the curve (i.e., measurement as *Di* → 0), ϕ is the slope of the curve, and θ is the inflection point of the curve (i.e., concentration giving a response half way between the top and bottom of the curve). The estimated inflection point of the curve was used as the drug response phenotype. That is, using these dose-response curves, we estimated the relative IC50 (effective dose that kills 50% of the cells) for MTT cell viability and the relative EC50 (concentration required to induce caspase 3/7 activity by 50%) for caspase3/7 activity. For simplicity of presentation, we will refer to these quantitative values as the IC50 for the MTT cytotoxicity assays and EC50 for the caspase 3/7 assays. We then applied rank-based inverse Gaussian transformation (i.e., Van de Waerden rank transformation). Summaries of the IC50 and EC50 values for both experimental batches are presented in Supplemental Table 1. We observed a high degree of correlation between many of the drug response phenotypes, as expected (Supplemental Figure 1).

### Genotyping and statistical methods

Germline DNA was genotyped on the Illumina Infinium 610K array, as previously described (Pharoah et al., 2013). All samples had genotype call rate >95% and were predicted by STRUCTURE (Pritchard et al., 2000) analysis to have greater than 80% European ancestry. SNPs were excluded with call rate < 95%, Hardy-Weinberg Equilibrium *p* < 10^−4^, or no variation in this set. Using the 1000 Genomes Project (Durbin et al., 2010) as reference, imputation was completed with *mach* and *minimac* in a two-step process (Howie et al., 2012), resulting in data on more than 30 million SNPs. Assessment of imputation quality was completed and high quality imputed markers (r^2^≥ 0.30 and MAF ≥ 0.01) were retained (6,243,550 SNPs).

The association of each SNP with *in vitro* drug response phenotypes IC50 or EC50 was evaluated with linear models using the expected genotype or “dosage” (i.e., additive or dose-response/trend model). Thus, a negative effect estimate indicates that the carriers of the minor/rare allele have lower IC50 (EC50) values (i.e., were more “sensitive” to treatment). The two *in vitro* experimental batches were analyzed separately followed by meta-analysis was conducted using *metal* (Willer et al., 2010), with weights applied for the number of samples in each group. We completed the GWAS analyses for each individual *in vitro* drug response phenotype, as opposed to a combined analysis with all phenotypes model together, due to the difference in mechanism of action between the drugs (i.e., not in the same drug class; Fridley et al., 2012). For annotation of results across gene regions, SNPs were mapped to genes within 2 KB using *Biofilter* (assembly CRCh37.p10, genome build 104.0; Bush et al., 2009). Pathway analysis used Ingenuity Pathway Analysis (IPA) (Ingenuity® Systems, www.ingenuity.com).

## Results

**Figure 1:**
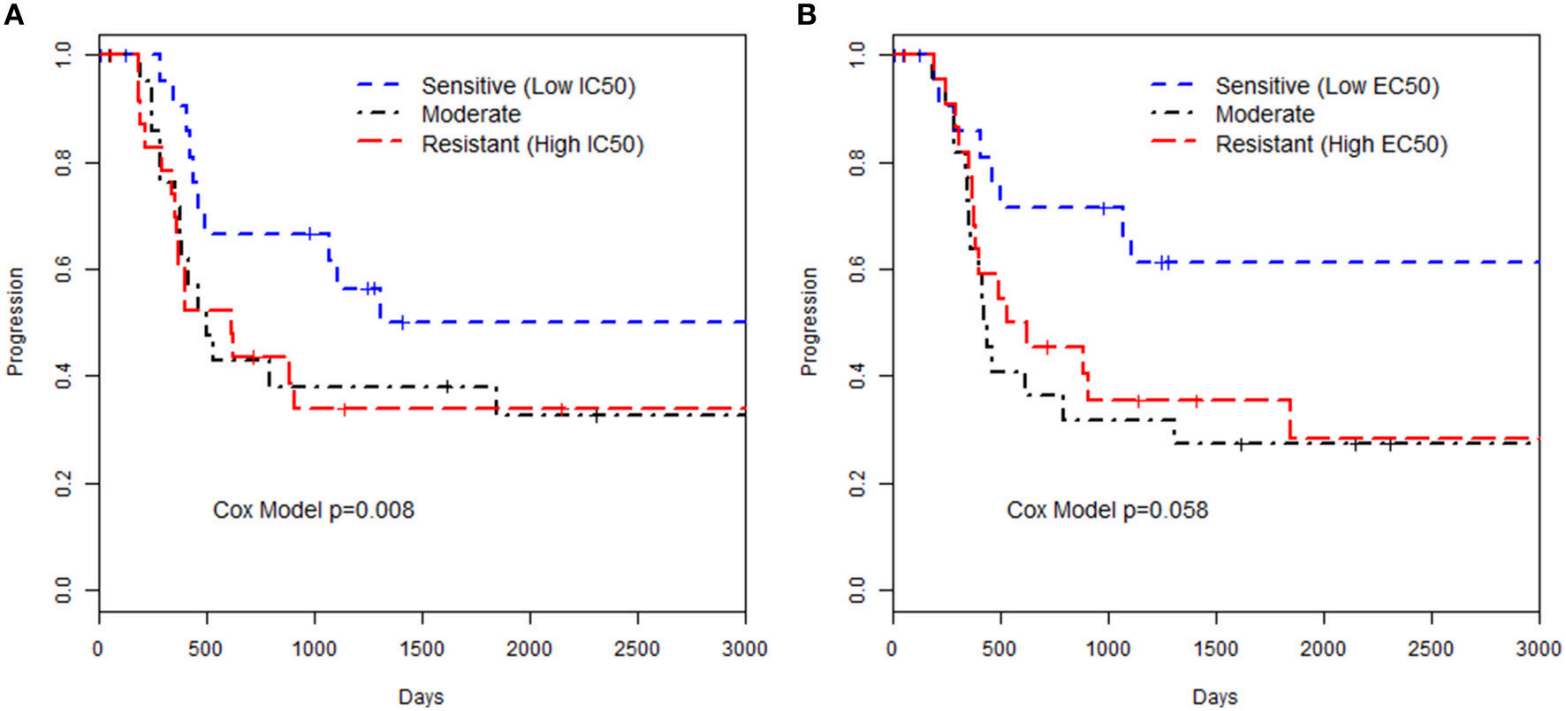
**Kaplan-Meier curves for time to progression and paclitaxel *in vitro* phenotypes**. The groupings were defined by 3 quartiles representing low, medium or high values for the phenotype based **(A)** MTT assay IC50 or **(B)** Caspase 3/7 EC50 assay. The *p*-value presented is from a Cox proportional hazards model with the *in vitro* phenotype modeled as a continuous measurement on the log-scale.

We examined the relationships of *in vitro* phenotypes with time to recurrence of EOC (40 of 74 patients had recurred or died). Of the 74 patients with LCLs included in this study, 51 had available information on the first two treatments used: 43 patients were treated with paclitaxel / carboplatin, 2 treated with paclitaxel/cisplatin, 3 treated with carboplatin/topotecan, and 3 treated with carboplatin/other less common agent. Paclitaxel *in vitro* drug response phenotypes were moderately associated with time to EOC recurrence (HR = 1.90 per unit increase in MTT IC50, *p* = 0.008; HR = 1.84 per unit increase in caspase 3/7 EC50, *p* = 0.058; Figure 1; Supplemental Table 2). This suggests that patients whose LCLs demonstrated greater sensitivity to the chemotherapeutics tested had improved outcome; as Figure 1 illustrates, LCLs that were sensitive to paclitaxel (as reflected by having low IC50 and low EC50 values) were from patients with longer time to progression as compared to patients with LCLs with high values. Although based on a small sample size, this provides, for the first time, a link between *in vitro* chemo-sensitivity testing and clinical outcome in EOC.

**Figure 2:**
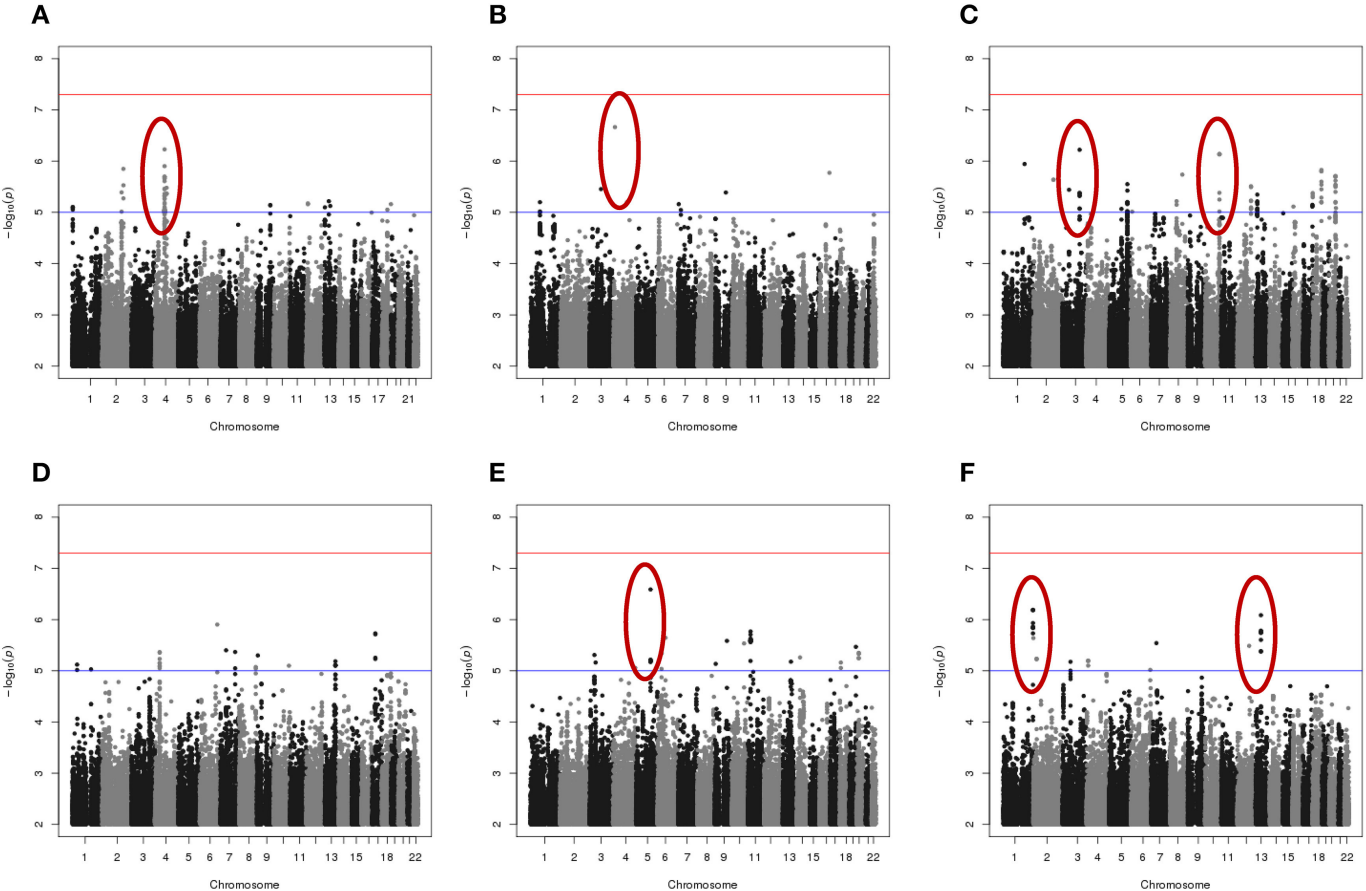
**Manhattan plots of the single SNP meta-analysis genome-wide association results based the six *in vitro* drug response measures among ovarian cancer patient LCLs. (A)** Carboplatin MTT; **(B)** Paclitaxel MTT; **(C)** Combination of carboplatin and paclitaxel MTT; **(D)** Carboplatin Caspase3/7; **(E)** Paclitaxel Caspase3/7; and **(F)** Combination of carboplatin and paclitaxel Caspase3/7. Blue line indicates *p* = 0.00001; Red line indicates *p* = 5 × 10^−8^. Highlighted regions (circled) are displayed in Figure 3.

**Figure 3:**
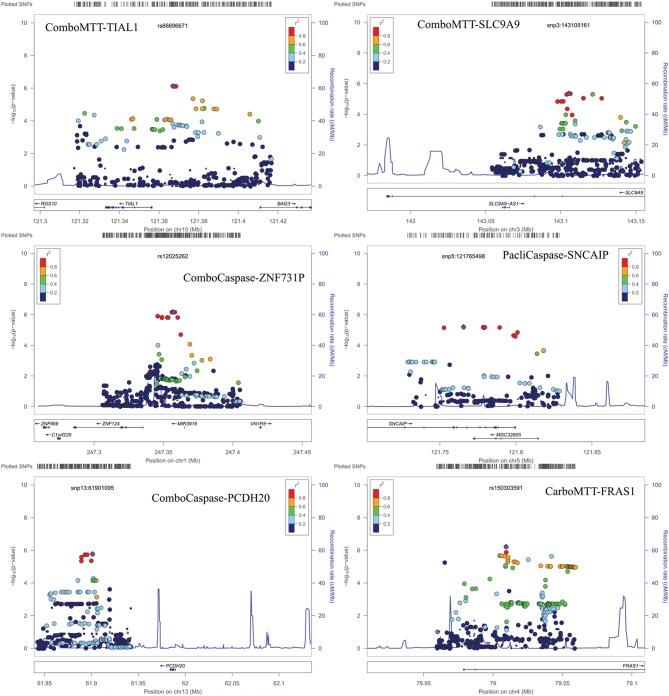
**Locus Zoom plots for regions with *p* < 10^−6^**. Note: BOD1L1 region not presented as only one SNP in region with *p* < 0.001.

Results of genome-wide association analyses for each drug response phenotype are presented in Figure 2. Regions with *p* < 10^−6^ are highlighted and are further displayed in Figure 3. Table 1 presents the SNPs associated with the drug response phenotype with *p* < 10^−6^. Overall, we found a greater proportion of significant results (e.g., at *p* < 10^−6^) for the combination therapy as compared to the single agent therapies. In particular, we found strong SNP associations with combination therapy in the following gene regions: *SLC9A9* (MAF = 0.41, *p* = 6 × 10^−7^), *TIAL1* (MAF = 0.23, *p* = 7.3 × 10^−7^), *ZNF731P* (MAF = 0.39, *p* = 6.6 × 10^−7^), and *PCDH20* (MAF = 0.42, *p* = 8.2 × 10^−7^). None of these regions were found to be moderately associated with single agent carboplatin or paclitaxel IC50 in other pharmacogenomic studies involving commercially available LCLs (Huang et al., 2011; Niu et al., 2012).

**Table 1:** **SNPs with *p* < 10^−6^ association with a drug response phenotype**.

**Drug**	**Phenotype**	**Nearest gene**	**SNP**	**Chr**	**Position**	**MAF**	**Meta-Analysis P**	**Group 1 (*****N*** = **33)**		**Group 2 (*****N*** = **41)**	
								**Estimate**	***P***	**Estimate**	***P***
Paclitaxel	MTT IC50	*BOD1L1*	rs185229225	4	13609129	0.02	2.2E-07	−2.53	8.0E-03	−11.64	4.7E-06
	Caspase 3/7 EC50	MGC32805/SNCAIP^*^	rs3842595	5	121778606	0.14	2.6E-07	−1.37	1.7E-03	−1.34	4.1E-05
Carboplatin	MTT IC50	*FRAS1*	rs150303591	4	79009309	0.29	5.9E-07	0.86	2.5E-03	1.02	6.3E-05
Combination	MTT IC50	SLC9A9^*^	rs201023017	3	143103669	0.41	6.0E-07	0.84	5.2E-04	0.70	3.3E-04
		*TIAL1*	rs66696671	10	121366953	0.23	7.3E-07	−0.89	2.9E-04	−0.76	6.6E-04
	Caspase 3/7 EC50	*ZNF731P*	rs12025262	1	247356732	0.39	6.6E-07	−0.84	2.5E-04	−0.71	6.9E-04
		*PCDH20*	rs10674174	13	61892075	0.42	8.2E-07	−0.73	5.3E-03	−0.86	3.7E-05

* *Nearest gene within 2000 base pairs*.

*For regions with multiple SNPs with p < 10^−6^, the most significant SNP is presented. A negative estimate indicates that carriers of the minor allele had, on average, lower IC50 or EC50 (“sensitive”) while a positive estimate indicates that carriers of the minor allele had, on average, higher IC50 or EC50 (“resistant”)*.

We evaluated potential overlap of loci associated with both phenotypes for a given drug. Only one SNP was found to be associated with a *p* < 10^−4^ with same direction of the effect for MTT IC50 and caspase3/7 EC50 values for paclitaxel, carboplatin, or combination treatment. An intronic SNP rs35067965 in *COLEC12* (chromosome 18, bp 455396) was associated with response to paclitaxel (MTT IC50 *p* = 2.2 × 10^−5^, caspase 3/7 EC50 *p* = 3.8 × 10^−5^; Table 2). We also examined overlap of associations at the level of genes, considering SNPs within 20 kb. This showed consistency of IC50 and EC50 results for paclitaxel response and *COLEC12* and revealed similar IC50 and EC50 associations for carboplatin response in the gene regions of *CTIF* and *CDH4*. As presented in Table 3, additional regions showed joint associations with response to multiple drugs, including variants in protein coding regions of *BRE, EML6, CTNNA2, LRP1B, EYS, NKAIN2, ANTXRL, COL13A1*, and *MTCL1* (SNPs in gene regions with *p* < 0.0001).

**Table 2:** **Gene regions with SNPs associated with both phenotypes for a given drug**.

**Drug**	**Gene**	**Phenotype**	SNP^*^	**Chr**	**Position**	***P***	**Direction^†^**
Paclitaxel	*COLEC12*	MTT IC50	rs35067965	18	455396	2.2E-05	−−
		Caspase 3/7 EC50	rs35067965	18	455396	3.8E-05	−−
Carboplatin	*CTIF*	MTT IC50	rs8091660	18	46087936	8.9E-06	−−
		Caspase 3/7 EC50	rs113867814	18	46259604	1.2E-05	−−
	*CDH4*	MTT IC50	rs2748151	20	60133486	4.7E-05	++
		Caspase 3/7 EC50	rs113594423	20	60379048	2.4E-05	++

* *Presenting most significant SNP in the region for the giving drug/phenotype. SNP within ± 20 KB of the listed gene*.

† *A negative estimate indicates that carriers of the minor/variant allele had, on average, lower IC50 or EC50 (“sensitive”) while a positive estimate indicates that carriers of the minor/variant allele had, on average, higher IC50 or EC50 (“resistant”)*.

**Table 3:** **Gene regions with SNPs associated with multiple drugs for any phenotype (*p* < 0.0001)**.

**Gene**	**Drug**	**Phenotype**	SNP^*^	**Chr**	**Position**	***P***	**Direction^†^**
*BRE*	Carboplatin	Caspase 3/7 EC50	rs5830067	2	28537890	1.7E-05	++
	Combination	Caspase 3/7 EC50	rs7572644	2	28320033	5.8E-06	−−
*EML6*	Paclitaxel	Caspase 3/7 EC50	rs75314082	2	55087315	7.9E-05	−−
	Combination	MTT IC50	rs17046344	2	55023600	4.9E-05	++
*LINC01122*	Paclitaxel	Caspase 3/7 EC50	rs72817940	2	58998563	6.4E-05	++
	Carboplatin	Caspase 3/7 EC50	rs4233974	2	59295043	2.6E-05	−−
*CTNNA2*	Carboplatin	MTT IC50	rs17261321	2	80197843	3.6E-05	++
	Combination	MTT IC50	rs6719499	2	80193386	6.0E-05	−−
*LRP1B*	Paclitaxel	MTT IC50	rs1525599	2	141778702	8.6E-05	++
	Combination	Caspase 3/7 EC50	rs13020675	2	142212928	6.2E-05	−−
*EYS*	Paclitaxel	Caspase 3/7 EC50	rs201083182	6	65736914	2.3E-06	−−
	Combination	Caspase 3/7 EC50	rs2064701	6	65676556	3.6E-05	++
*NKAIN2*	Paclitaxel	Caspase 3/7 EC50	rs550987	6	124905510	4.1E-05	−−
	Combination	Caspase 3/7 EC50	rs670616	6	124885773	7.8E-05	++
*C7orf65*	Carboplatin	Caspase 3/7 EC50	rs10230114	7	47705506	2.4E-05	++
	Combination	Caspase 3/7 EC50	rs11771997	7	47712495	2.4E-05	++
*ANTXRL*	Paclitaxel	Caspase 3/7 EC50	rs12572446	10	47665906	4.3E-05	++
	Combination	Caspase 3/7 EC50	rs10906942	10	47670851	4.9E-05	++
*COL13A1*	Carboplatin	Caspase 3/7 EC50	rs10999018	10	71654602	2.4E-05	++
	Combination	Caspase 3/7 EC50	rs77535242	10	71652985	3.5E-05	++
*TMEM132D*	Paclitaxel	Caspase 3/7 EC50	rs77438645	12	130304313	−−	−−
	Carboplatin	Caspase 3/7 EC50	rs1451904	12	130166947	6.5E-05	++
*MTCL1*	Carboplatin	Caspase 3/7 EC50	rs690089	18	8845223	7.8E-05	−−
	Combination	Caspase 3/7 EC50	rs35765215	18	8839469	6.0E-05	−−

* *Presenting most significant SNP in the region for the giving drug/phenotype. SNP within ± 20 KB of the listed gene*.

† *A negative estimate indicates that carriers of the minor/variant allele had, on average, lower IC50 or EC50 (“sensitive”) while a positive estimate indicates that carriers of the minor/variant allele had, on average, higher IC50 or EC50 (“resistant”)*.

Because of the suggested association between *in vitro* paclitaxel MTT IC50 response and time to EOC recurrence (Supplemental Table 2), we also examined recurrence association with SNPs rs185229225 (intronic *BOD1L1*) rs35067965 (intronic *COLEC12*) and rs1525599 (intronic *LRP1B*) which were associated with paclitaxel MTT IC50 (Tables 1–3, respectively). However, none of these SNPs were associated with time to recurrence with a nominal *p* < 0.05 (data not shown).

## Discussion

In this proof of concept study, we explored use of LCLs derived from EOC patients followed for clinical response as a model for discovery of pharmacogenomics markers. LCLs were treated with varying concentration of the chemotherapeutics agents (carboplatin and paclitaxel and, uniquely, their combination) that were used for the treatment and cellular chemo-sensitivity was determined by measuring cell viability and activation of caspase activity (as a marker of apoptosis) post drug treatment. Genome-wide association studies were performed to identify inherited markers associated with these measures of *in vitro* chemo-sensitivity (i.e., MTT IC50 and caspase 3/7 EC50 values) and the relationships between *in vitro* measures and clinical outcome was explored.

Although the sample size was small limiting the power of the study, some of the implicated biologically interesting genes are worthy of discussion. Pathway analysis of genes with SNPs showing association with one of the drug response phenotypes (at *p* < 10^−6^), both phenotypes for a given drug (at *p* < 10^−4^), or multiple drugs for any phenotype (at *p* < 10^−4^) found enrichment in genes related to malignant solid tumor and epithelial cancers (Supplemental Figure 2A). Among the top canonical pathways represented by these genes were “Epithelial Adherens Junction Signaling,” “Sertoli Cell Junction Signaling,” and “Endometrial Cancer Signaling” (Supplemental Figure 2B).

In addition, genes such as *CTNNA2* and *CDH4*, both tumor suppressor genes with role in cell adhesion were found to be associated with chemo-sensitivity in carboplatin alone or combination treatments. SNPs in *CTNNA2*- catenin (cadherin-associated protein) alpha2, a structural constituent of cytoskeleton and cadherin binding was associated with *in vitro* cytotoxicity to carboplatin alone as well as in combination with paclitaxel. CTNNA2 has been shown to be frequently mutated in laryngeal carcinomas with mutations predictive of poor prognosis (Fanjul-Fernandez et al., 2013). Additionally, SNPs within *CTNNA2* have recently been implicated in breast cancer (Haryono et al., 2015) and its role in tumor progression and metastasis has been suggested for multiple cancers (Mcgranahan et al., 2015). Variants in *CTNNA2* have also been implicated in schizophrenia (Mexal et al., 2008) and alcohol addiction (Song and Zhang, 2014). CTNNA2 SNPs associated with carboplatin and paclitaxel MTT IC50 were both intronic and present functional relevance of these is not known. *CDH4*, codes for cadherin, and has been implicated in nasopharyngeal carcinoma (Du et al., 2011) and aberrant methylation of CDH4 promoter has been colorectal and gastric cancer (Miotto et al., 2004). Our results identified two intronic SNPs (rs2748151 and rs113594423) that were associated with carboplatin resistant as measured by cell death (IC50) and apoptosis (caspase 3/7 EC50). Variants in *PCDH20*, another member of cadherin family, were also found to be associated *in vitro* drug response. *PCHD20* codes for protocadherin20 and functions as a tumor suppressor by interacting with Wnt/b-catenin signaling (Chen et al., 2015; Lv et al., 2015).

Another gene with role in cell adhesion identified in our study was *FRAS1*, which encodes for an extracellular matrix protein and is involved in the regulation of epidermal-basement membrane adhesion and organogenesis during development. Inherited mutations in *FRAS1*, and *FREM2*, have been associated with development of Fraser syndrome. *FRAS1* has also been implicated in ERK signaling and influence migration and invasion of lung cancer cell line by influencing FAK signaling (Zhan et al., 2014), suggesting its role in tumorigenesis and metastasis of lung cancer. Although the genes described above are involved cell adhesion/cell migration, the functional significance of the intronic SNPs identified in this study is not known and would require further investigation.

Two intronic variants within *BRE* were found to be associated with caspase 3/7 levels for carboplatin and combination therapy (indel rs5830067 and rs7572664). *BRE* encodes for Brain and reproductive Organ-Expressed (TNFRSF1A modulator) and is a component of BRCA1-A DNA damage repair complex that recognizes Lys 62linked ubiquitinated H2A and H2Ax at DNA lesions, resulting in recruitment of BRCA1-BARD1 to double strand DNA breaks (Li et al., 1995). *BRE* expression has been shown to be predictive of disease free survival in non-familial breast cancer patients (Noordermeer et al., 2012) and recent studies show its involvement in both intrinsic and extrinsic apoptotic pathways by influencing *XIAP* (Chui et al., 2014). Variation within *EML6*, which is involved assembly dynamics of microtubules, was found to be associated with platinum-sensitivity which was of interest since paclitaxel's mechanism of action involved disruption of microtubules; however no evidence exists in the literature on functional relevance of these particular SNPs within *EML6*. No other genes involved in microtubule protein were identified with respect to paclitaxel chemo-sensitivity.

Lastly, two intronic variants within *LRP1B* (low density lipoprotein related protein 1B) were associated with paclitaxel and combination therapy drug response phenotypes. *LRP1B* is a tumor suppressor with decreased expression in several primary cancers and is among 10 most significantly deleted genes across 3312 cancer samples (Langbein et al., 2002; Sonoda et al., 2004; Nakagawa et al., 2006; Prazeres et al., 2011). In renal cell cancer, down-regulation of *LRP1B* has been shown to regulate cell motility and actin cytoskeleton reorganization (Lu et al., 2013). Germline SNPs/ haplotype in *LRP1B* have been associated with aging without cognitive decline (Poduslo et al., 2010); however, associations of germline SNPs with incidence/progression of cancer and pharmacogenomics have yet to be reported.

In summary, using a patient-derived cell-based model system to generate several *in vitro* drug response phenotypes on a clinically followed set of EOC cases we have identified genetic loci associated with response to platinum-taxane therapies. Overall our results identified germ-line SNPs in multiple cell adhesion molecules and several tumor suppressor genes (*PCDH20, LRP1B, CDH4*, and *CTNNA2*). However, none of the most associated SNPs were reported by Huang et al. (2011) or associated with mRNA gene expression in lymphoblastoid cell lines (http://www.ncbi.nlm.nih.gov/projects/gap/eqtl/index.cgi). Further studies are needed to determine if these SNPs are truly associated with drug response or if they represent false-positive findings. Similar to other studies comparing *in vitro* chemo-sensitivity with clinical outcomes (Huang et al., 2011; Gamazon et al., 2013), our findings suggest that *in vitro* response to paclitaxel correlates with time to disease recurrence indicating that this model may have utility in several types of future studies. On possible explanation for the observation that paclitaxel correlates with recurrence and not carboplatin may be the fact that the majority of EOC patients eventually develop platinum resistant tumors and the main factor related to future response maybe attributed to response to taxane therapy. Further research is needed to understand the mechanism by which genomic loci impact clinical response in ovarian cancer patients to the most common regimen used in the treatment of ovarian cancer following surgery.

## Author contributions

Conceived and designed the study: BF, JL, EG. Collected cell lines and clinical information: EG. Genotyping data: EG, BF. Performed drug assays: TG, JL. Statistical Analyses: RR, JD, AW, BF. Wrote the paper: BF, JL, EG. Reviewed Manuscript: ALL.

## Funding

Funding for this research was provided by National Institute of Health (R01 CA122443, P50 CA136393, P30 CA168524, P30 CA15083, P20 GM103418, R21 CA182715), the Minnesota Partnership, the Fraternal Order of Eagles Cancer Research Fund, and the Minnesota Ovarian Cancer Alliance.

### Conflict of interest statement

The authors declare that the research was conducted in the absence of any commercial or financial relationships that could be construed as a potential conflict of interest. The reviewer (EG-M) and Handling Editor declared their shared affiliation, and the Handling Editor states that the process nevertheless met the standards of a fair and objective review.

## References

[B1] Bergmann T. K. Brasch-Andersen C. Green H. Mirza M. Pedersen R. S. Nielsen F.. (2011). Impact of CYP2C8^*^3 on paclitaxel clearance: a population pharmacokinetic and pharmacogenomic study in 93 patients with ovarian cancer. Pharmacogenomics J. 11, 113–120. 10.1038/tpj.2010.19 20368717

[B2] Bush W. S. Dudek S. M. Ritchie M. D. (2009). Biofilter: a knowledge-integration system for the multi-locus analysis of genome-wide association studies. Pac. Symp. Biocomput. 14, 368–379. 10.1142/9789812836939_0035 19209715 PMC2859610

[B3] Chen T. Long B. Ren G. Xiang T. Li L. Wang Z.. (2015). *Protocadherin20* acts as a tumor suppressor gene: epigenetic inactivation in nasopharyngeal carcinoma. J. Cell. Biochem. 116, 1766–1775. 10.1002/jcb.25135 25736877

[B4] Chui Y. L. Ma C. H. Li W. Xu Z. Yao Y. Lin F. K.. (2014). Anti-apoptotic protein BRE/BRCC45 attenuates apoptosis through maintaining the expression of caspase inhibitor XIAP in mouse Lewis lung carcinoma D122 cells. Apoptosis 19, 829–840. 10.1007/s10495-013-0963-y 24395041

[B5] Dekou V. Whincup P. Papacosta O. Ebrahim S. Lennon L. Ueland P. M.. (2001). The effect of the C677T and A1298C polymorphisms in the methylenetetrahydrofolate reductase gene on homocysteine levels in elderly men and women from the British regional heart study. Atherosclerosis 154, 659–666. 10.1016/S0021-9150(00)00522-0 11257267

[B6] Du C. Huang T. Sun D. Mo Y. Feng H. Zhou X.. (2011). CDH4 as a novel putative tumor suppressor gene epigenetically silenced by promoter hypermethylation in nasopharyngeal carcinoma. Cancer Lett. 309, 54–61. 10.1016/j.canlet.2011.05.016 21665361

[B7] Durbin R. M. Abecasis G. R. Altshuler D. L. Auton A. Brooks L. D. Durbin R. M.. (2010). A map of human genome variation from population-scale sequencing. Nature 467, 1061–1073. 10.1038/nature09534 20981092 PMC3042601

[B8] Fanjul-Fernandez M. Quesada V. Cabanillas R. Cadinanos J. Fontanil T. Obaya A.. (2013). Cell-cell adhesion genes CTNNA2 and CTNNA3 are tumour suppressors frequently mutated in laryngeal carcinomas. Nat. Commun. 4, 2531. 10.1038/ncomms3531 24100690

[B9] Fridley B. L. Jenkins G. D. Batzler A. Wang L. Ji Y. Li F.. (2012). Multivariate models to detect genomic signatures for a class of drugs: application to thiopurines pharmacogenomics. Pharmacogenomics J. 12, 105–110. 10.1038/tpj.2010.83 21060324 PMC3084322

[B10] Gamazon E. R. Huang R. S. Cox N. J. Dolan M. E. (2010). Chemotherapeutic drug susceptibility associated SNPs are enriched in expression quantitative trait loci. Proc. Natl. Acad. Sci. U.S.A. 107, 9287–9292. 10.1073/pnas.1001827107 20442332 PMC2889115

[B11] Gamazon E. R. Lamba J. K. Pounds S. Stark A. L. Wheeler H. E. Cao X.. (2013). Comprehensive genetic analysis of cytarabine sensitivity in a cell-based model identifies polymorphisms associated with outcome in AML patients. Blood 121, 4366–4376. 10.1182/blood-2012-10-464149 23538338 PMC3663430

[B12] Garcia-Martin E. Pizarro R. M. Martinez C. Gutierrez-Martin Y. Perez G. Jover R.. (2006). Acquired resistance to the anticancer drug paclitaxel is associated with induction of cytochrome P450 2C8. Pharmacogenomics 7, 575–585. 10.2217/14622416.7.4.575 16753005

[B13] Haryono S. J. Datasena I. G. Santosa W. B. Mulyarahardja R. Sari K. (2015). A pilot genome-wide association study of breast cancer susceptibility loci in Indonesia. Asian Pac. J. Cancer Prev. 16, 2231–2235. 10.7314/APJCP.2015.16.6.2231 25824743

[B14] Hertz D. L. Motsinger-Reif A. A. Drobish A. Winham S. J. Mcleod H. L. Carey L. A.. (2012). CYP2C8^*^3 predicts benefit/risk profile in breast cancer patients receiving neoadjuvant paclitaxel. Breast Cancer Res. Treat. 134, 401–410. 10.1007/s10549-012-2054-0 22527101 PMC3727245

[B15] Hewett M. Oliver D. E. Rubin D. L. Easton K. L. Stuart J. M. Altman R. B.. (2002). PharmGKB: the Pharmacogenetics Knowledge Base. Nucleic Acids Res. 30, 163–165. 10.1093/nar/30.1.163 11752281 PMC99138

[B16] Howie B. Fuchsberger C. Stephens M. Marchini J. Abecasis G. R. (2012). Fast and accurate genotype imputation in genome-wide association studies through pre-phasing. Nat. Genet. 44, 955–959. 10.1038/ng.2354 22820512 PMC3696580

[B17] Huang R. S. Johnatty S. E. Gamazon E. R. Im H. K. Ziliak D. Duan S.. (2011). Platinum sensitivity-related germline polymorphism discovered via a cell-based approach and analysis of its association with outcome in ovarian cancer patients. Clin. Cancer Res. 17, 5490–5500. 10.1158/1078-0432.CCR-11-0724 21705454 PMC3160494

[B18] Huizing M. T. Misser V. H. Pieters R. C. Ten Bokkel Huinink W. W. Veenhof C. H. Vermorken J. B.. (1995). Taxanes: a new class of antitumor agents. Cancer Invest. 13, 381–404. 10.3109/07357909509031919 7627725

[B19] Johnatty S. E. Beesley J. Gao B. Chen X. Lu Y. Law M. H.. (2013). ABCB1 (MDR1) polymorphisms and ovarian cancer progression and survival: a comprehensive analysis from the Ovarian Cancer Association Consortium and The Cancer Genome Atlas. Gynecol. Oncol. 131, 8–14. 10.1016/j.ygyno.2013.07.107 23917080 PMC3795832

[B20] Jordan M. A. Wilson L. (2004). Microtubules as a target for anticancer drugs. Nat. Rev. Cancer 4, 253–265. 10.1038/nrc1317 15057285

[B21] Langbein S. Szakacs O. Wilhelm M. Sukosd F. Weber S. Jauch A.. (2002). Alteration of the LRP1B gene region is associated with high grade of urothelial cancer. Lab. Invest. 82, 639–643. 10.1038/labinvest.3780458 12004004

[B22] Li F. Fridley B. L. Matimba A. Kalari K. R. Pelleymounter L. Moon I.. (2010). Ecto-5'-nucleotidase and thiopurine cellular circulation: association with cytotoxicity. Drug Metab. Dispos. 38, 2329–2338. 10.1124/dmd.110.035220 20855458 PMC2993460

[B23] Li L. Fridley B. Kalari K. Jenkins G. Batzler A. Safgren S.. (2008). Gemcitabine and cytosine arabinoside cytotoxicity: association with lymphoblastoid cell expression. Cancer Res. 68, 7050–7058. 10.1158/0008-5472.CAN-08-0405 18757419 PMC2562356

[B24] Li L. Yoo H. Becker F. F. Ali-Osman F. Chan J. Y. (1995). Identification of a brain- and reproductive-organs-specific gene responsive to DNA damage and retinoic acid. Biochem. Biophys. Res. Commun. 206, 764–774. 10.1006/bbrc.1995.1108 7826398

[B25] Lu G. Zhang G. Zhang C. Chen C. Liu R. (2013). A study of 131iodine-labeling of histamine-indomethacin: its *in vivo* therapeutic effect and anti-tumor mechanisms in Lewis-bearing lung cancer. Radiat. Oncol. 8:74. 10.1186/1748-717X-8-74 23531319 PMC3627608

[B26] Lv J. Zhu P. Yang Z. Li M. Zhang X. Cheng J.. (2015). PCDH20 functions as a tumour-suppressor gene through antagonizing the Wnt/beta-catenin signalling pathway in hepatocellular carcinoma. J. Viral Hepat. 22, 201–211. 10.1111/jvh.12265 24910204 PMC4344823

[B27] Marsh S. (2009). Pharmacogenomics of taxane/platinum therapy in ovarian cancer. Intern. J. Gynecol. Cancer 19 (Suppl. 2), S30–S34. 10.1111/igc.0b013e3181c10513 19955911

[B28] Martinez C. Garcia-Martin E. Pizarro R. M. Garcia-Gamito F. J. Agundez J. A. (2002). Expression of paclitaxel-inactivating CYP3A activity in human colorectal cancer: implications for drug therapy. Br. J. Cancer 87, 681–686. 10.1038/sj.bjc.6600494 12237780 PMC2364247

[B29] Mcgranahan N. Favero F. De Bruin E. C. Birkbak N. J. Szallasi Z. Swanton C. (2015). Clonal status of actionable driver events and the timing of mutational processes in cancer evolution. Sci. Transl. Med. 7, 283ra254. 10.1126/scitranslmed.aaa1408 25877892 PMC4636056

[B30] Mexal S. Berger R. Pearce L. Barton A. Logel J. Adams C. E.. (2008). Regulation of a novel alphaN-catenin splice variant in schizophrenic smokers. Am. J. Med. Genet. B Neuropsychiatr. Genet. 147B, 759–768. 10.1002/ajmg.b.30679 18163523 PMC2701353

[B31] Miotto E. Sabbioni S. Veronese A. Calin G. A. Gullini S. Liboni A.. (2004). Frequent aberrant methylation of the CDH4 gene promoter in human colorectal and gastric cancer. Cancer Res. 64, 8156–8159. 10.1158/0008-5472.CAN-04-3000 15548679

[B32] Nakagawa T. Pimkhaokham A. Suzuki E. Omura K. Inazawa J. Imoto I. (2006). Genetic or epigenetic silencing of low density lipoprotein receptor-related protein 1B expression in oral squamous cell carcinoma. Cancer Sci. 97, 1070–1074. 10.1111/j.1349-7006.2006.00283.x 16918994 PMC11159176

[B33] Niu N. Qin Y. Fridley B. L. Hou J. Kalari K. R. Zhu M.. (2010). Radiation pharmacogenomics: a genome-wide association approach to identify radiation response biomarkers using human lymphoblastoid cell lines. Genome Res. 20, 1482–1492. 10.1101/gr.107672.110 20923822 PMC2963812

[B34] Niu N. Schaid D. J. Abo R. P. Kalari K. Fridley B. L. Feng Q.. (2012). Genetic association with overall survival of taxane-treated lung cancer patients - a genome-wide association study in human lymphoblastoid cell lines followed by a clinical association study. BMC Cancer 12:422. 10.1186/1471-2407-12-422 23006423 PMC3573965

[B35] Noordermeer S. M. Wennemers M. Bergevoet S. M. Van Der Heijden A. Tonnissen E. Sweep F. C.. (2012). Expression of the BRCA1 complex member BRE predicts disease free survival in breast cancer. Breast Cancer Res. Treat. 135, 125–133. 10.1007/s10549-012-2122-5 22706632 PMC3413819

[B36] Peethambaram P. Fridley B. L. Vierkant R. A. Larson M. C. Kalli K. R. Elliott E. A.. (2011). Polymorphisms in ABCB1 and ERCC2 associated with ovarian cancer outcome. Int. J. Mol. Epidemiol. Genet. 2, 185–195. 21686133 PMC3110393

[B37] Pharoah P. D. Tsai Y. Y. Ramus S. J. Phelan C. M. Goode E. L. Lawrenson K.. (2013). GWAS meta-analysis and replication identifies three new susceptibility loci for ovarian cancer. Nat. Genet. 45, 362–370, 370e361-362. 10.1038/ng.2564 23535730 PMC3693183

[B38] Poduslo S. E. Huang R. Spiro A. III. (2010). A genome screen of successful aging without cognitive decline identifies LRP1B by haplotype analysis. Am. J. Med. Genet. B Neuropsychiatr. Genet. 153B, 114–119. 10.1002/ajmg.b.30963 19367585

[B39] Prazeres H. Torres J. Rodrigues F. Pinto M. Pastoriza M. C. Gomes D.. (2011). Chromosomal, epigenetic and microRNA-mediated inactivation of LRP1B, a modulator of the extracellular environment of thyroid cancer cells. Oncogene 30, 1302–1317. 10.1038/onc.2010.512 21057533

[B40] Pritchard J. K. Stephens M. Donnelly P. (2000). Inference of population structure using multilocus genotype data. Genetics 155, 945–959. 10835412 10.1093/genetics/155.2.945 PMC1461096

[B41] Rodriguez-Antona C. (2010). Pharmacogenomics of paclitaxel. Pharmacogenomics 11, 621–623. 10.2217/pgs.10.32 20415548

[B42] Siegel R. L. Miller K. D. Jemal A. (2015). Cancer statistics, 2015. CA Cancer J. Clin. 65, 5–29. 10.3322/caac.21254 25559415

[B43] Song C. Zhang H. (2014). TARV: tree-based analysis of rare variants identifying risk modifying variants in CTNNA2 and CNTNAP2 for alcohol addiction. Genet. Epidemiol. 38, 552–559. 10.1002/gepi.21843 25041903 PMC4154634

[B44] Sonoda I. Imoto I. Inoue J. Shibata T. Shimada Y. Chin K.. (2004). Frequent silencing of low density lipoprotein receptor-related protein 1B (LRP1B) expression by genetic and epigenetic mechanisms in esophageal squamous cell carcinoma. Cancer Res. 64, 3741–3747. 10.1158/0008-5472.CAN-04-0172 15172977

[B45] White K. L. Vierkant R. A. Fogarty Z. C. Charbonneau B. Block M. S. Pharoah P. D. Goode, E.L.. (2013). Analysis of over 10,000 Cases finds no association between previously reported candidate polymorphisms and ovarian cancer outcome. Cancer Epidemiol. Biomarkers Prev. 22, 987–992. 10.1158/1055-9965.EPI-13-0028 23513043 PMC3650102

[B46] Willer C. J. Li Y. Abecasis G. R. (2010). METAL: fast and efficient meta-analysis of genomewide association scans. Bioinformatics 26, 2190–2191. 10.1093/bioinformatics/btq340 20616382 PMC2922887

[B47] Wu T. Y. Fridley B. L. Jenkins G. D. Batzler A. Wang L. Weinshilboum R. M. (2011). Mycophenolic acid response biomarkers: a cell line model system-based genome-wide screen. Int. Immunopharmacol. 11, 1057–1064. 10.1016/j.intimp.2011.02.027 21396482 PMC3138818

[B48] Zhan Q. Huang R. F. Liang X. H. Ge M. X. Jiang J. W. Lin H.. (2014). FRAS1 knockdown reduces A549 cells migration and invasion through downregulation of FAK signaling. Int. J. Clin. Exp. Med. 7, 1692–1697. 25126166 PMC4132130

