# Supplementary material for: Genome-Wide Study of Response to Platinum, Taxane, and Combination Therapy in Ovarian Cancer: In vitro Phenotypes, Inherited Variation, and Disease Recurrence
Source: Front Genet. 2016 Mar 22;7:37. doi: 10.3389/fgene.2016.00037 (PMC4801852; doi:10.3389/fgene.2016.00037)

Response to platinum, taxane, and combination therapy in ovarian cancer: in vitro phenotypes, inherited variation, and disease recurrence

Brooke L. Fridley, Taraswi Mitra Ghosh, Alice Wang, Rama Raghavan, Junqiang Dai, Ellen L. Goode, Jatinder K. Lamba

Supplemental Table 1: Mean (SD) *in vitro* phenotype measures collected on LCLs from 74 EOC patients and comparison across experimental batch

**Phenotype**	**Treatment**	**Batch 1 (N=33)**	**Batch 2 (N=41)**	**P***
MTT IC50	Paclitaxel	14.22 (8.4)	27.66 (55.1)	0.131
	Carboplatin	55.16 (73.9)	105.52 (138.0)	0.049
	Combination	31.10 (37.6)	47.16 (84.8)	0.281
Caspase 3/7 EC50	Paclitaxel	11.34 (6.7)	9.39 (6.2)	0.865
	Carboplatin	26.00 (13.9)	26.58 (15.3)	0.203
	Combination	10.36 (4.39)	10.28 (5.9)	0.953

*P is result from testing difference in two groups based on a two-sample t-test assuming unequal variances (Welch Test)

Supplemental Table 2: Hazard ratios (p-value) associating *in vitro* drug phenotypes with time to disease recurrence

	Caspase 3/7 EC50	MTT IC50
Paclitaxel	1.84 (**0.058**)	1.90 (**0.008**)
Carboplatin	1.00 (0.991)	1.00 (1.000)
Combination	1.42 (0.376)	1.01 (0.930)

* P-value determined from a likelihood ratio test; Hazard Ratio > 1 indicates worse outcome for subjects with high IC50 ("resistant"); 40 events; bold, p<0.10

Supplemental Figure 1: Scatterplot matrix of drug response phenotypes by experimental batch. Spearman correlations are presented on the upper panel of each plot (log scale). (A) Batch 1 (N = 33), the highest correlation being between paclitaxel IC50 and combination IC50 (r = 0.81) and caspase EC50 measurements for paclitaxel and combination treatments (r = 0.78). (B) Batch 2 (N = 41), the highest correlation was between paclitaxel and combination IC50 measurements (r = 0.39).

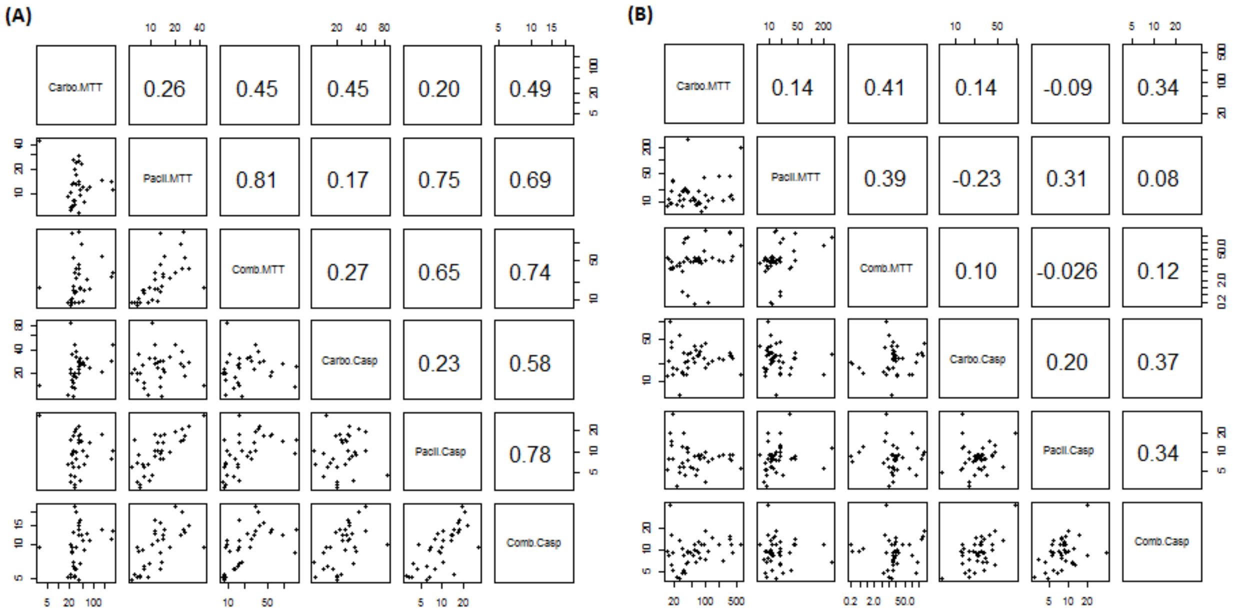

Supplemental Figure 2: Pathway analysis of genes with SNPs that demonstrated significant association with LCL chemosensitivity with carboplatin or paclitaxel alone or in combination. A) Significant number of genes mapped to malignant solid tumor or epithelial cancer; B) Top canonical pathways and interaction network among the pathways represented by significantly associated genes.

(A)

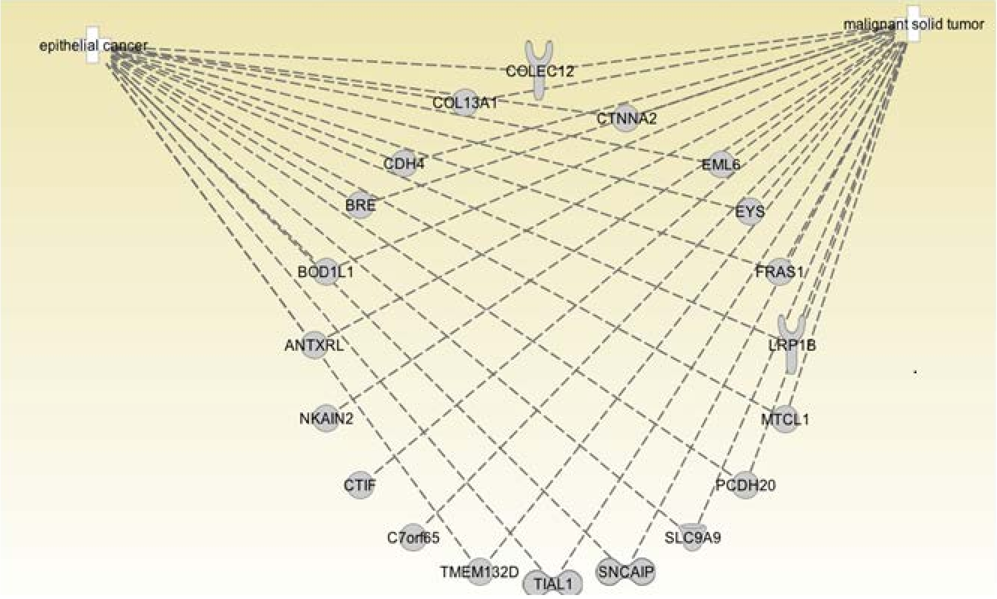

(B)

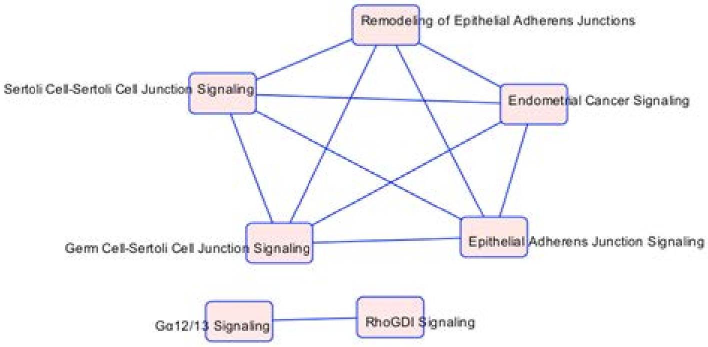

Supplement: Supplementary file 1 [file DataSheet1.PDF]
